# Weighted Single-Step Genomic Best Linear Unbiased Prediction Method Application for Assessing Pigs on Meat Productivity and Reproduction Traits

**DOI:** 10.3390/ani12131693

**Published:** 2022-06-30

**Authors:** Artem Kabanov, Ekaterina Melnikova, Sergey Nikitin, Maria Somova, Oleg Fomenko, Valeria Volkova, Olga Kostyunina, Tatiana Karpushkina, Elena Martynova, Elena Trebunskikh

**Affiliations:** 1 L.K. Ernst Federal Research Center for Animal Husbandry, Dubrovitzy Estate, Podolsk District, 142132 Moscow, Russia; melnikovaee_1982@inbox.ru (E.M.); snikitin@vij.ru (S.N.); somova-mm@yandex.ru (M.S.); moonlit_elf@mail.ru (V.V.); kostolan@yandex.ru (O.K.); tati.kriz@gmail.com (T.K.); 2 All-Russian Dairy Research Institute, 115093 Moscow, Russia; fomenych@rambler.ru; 3 Center of Life Sciences, Skolkovo Institute of Science and Technology, 143026 Moscow, Russia; elenamartynovaster@gmail.com; 4 LLC “TOPGEN”, Verkhnyaya Khava, 396110 Voronezh, Russia; terramio7@mail.ru

**Keywords:** genomic evaluation, single-step GBLUP, weighted single-step GBLUP, pigs, SNP effects, estimation reliability, validation of genomic estimates

## Abstract

**Simple Summary:**

The effectiveness of genomic selection in pig breeding depends largely on genomic prediction accuracy. The relatively short generation interval and the high selection intensity make the issue of the selection criterion accuracy extremely important. Genotyping animals by SNP markers makes it possible to increase the accuracy of breeding criteria for young replacement animals. However, the applied computational algorithm plays an equally important role, that is, the method of calculating the genomic estimates. The study compared three methods (BLUP AM, ssGBLUP, wssGBLUP) for the assessment of pigs’ breeding value according to five main breeding traits.

**Abstract:**

Changes in the accuracy of the genomic estimates obtained by the ssGBLUP and wssGBLUP methods were evaluated using different reference groups. The weighting procedure’s reasonableness of application Pwas considered to improve the accuracy of genomic predictions for meat, fattening and reproduction traits in pigs. Six reference groups were formed to assess the genomic data quantity impact on the accuracy of predicted values (groups of genotyped animals). The datasets included 62,927 records of meat and fattening productivity (fat thickness over 6–7 ribs (BF1, mm)), muscle depth (MD, mm) and precocity up to 100 kg (age, days) and 16,070 observations of reproductive qualities (the number of all born piglets (TNB) and the number of live-born piglets (NBA), according to the results of the first farrowing). The wssGBLUP method has an advantage over ssGBLUP in terms of estimation reliability. When using a small reference group, the difference in the accuracy of ssGBLUP over BLUP AM is from −1.9 to +7.3 percent points, while for wssGBLUP, the change in accuracy varies from +18.2 to +87.3 percent points. Furthermore, the superiority of the wssGBLUP is also maintained for the largest group of genotyped animals: from +4.7 to +15.9 percent points for ssGBLUP and from +21.1 to +90.5 percent points for wssGBLUP. However, for all analyzed traits, the number of markers explaining 5% of genetic variability varied from 71 to 108, and the number of such SNPs varied depending on the size of the reference group (79–88 for BF1, 72–81 for MD, 71–108 for age). The results of the genetic variation distribution have the greatest similarity between groups of about 1000 and about 1500 individuals. Thus, the size of the reference group of more than 1000 individuals gives more stable results for the estimation based on the wssGBLUP method, while using the reference group of 500 individuals can lead to distorted results of GEBV.

## 1. Introduction

The improvement of methods for assessing the farm animals breeding values has two main directions: attracting as much data about the animals and their relatives as possible and developing methods that allow the more efficient use of available information. Thus, genomic evaluation methods have significantly improved the prediction accuracy of the individual genetic value by using marker genotype data [1,2,3]. The next step in this process was the development of mathematical procedures ensuring the most reasonable application of this information and special algorithms to reduce computational and memory costs, in particular, the determination of the optimal weight of the data involved [4,5]. The density of SNP marker distributions over the genome greatly impacts genomic prediction accuracy [6]. Applying the WGS strategy seems the most preferable for genomic selection because microarray chips cover only a limited number of individual SNPs, while whole-genome sequencing provides information over the entire genome [7,8]. However, the genomic evaluation based on microarray genotyping data is still most widespread due to having the best cost-benefit ratio.

The primary assumption of genomic assessment methods based on linear mixed models is that all markers have the same contribution to the overall genetic variability of a given trait. However, it is difficult to agree that such a situation occurs [9,10]. The ability to account for individual SNP marker weight in genomic estimation has led to the rise of the weighted single-step genomic BLUP (wssGBLUP) methodology, which is able to differentiate the contribution of every single marker effect to the trait variability [11]. A large number of studies are devoted to searching quantitative trait loci and casual genes, the identification of which could significantly reduce the marker panels and improve the individual genetic prediction accuracy. In this regard, many investigations have analyzed the associations between individual SNP markers and/or their positional successions (also known as «windows») and the phenotype variability of key breeding traits in livestock [12]. The associated study results, such as identifying individual genes and large chromosomal segments obtained by different research groups, are often not entirely consistent with each other. This is due to several objective and subjective reasons: trait polygenicity, low marker effect values, the insufficient information content of traditional phenotypic measurements, the significant influence of paratype factors on the phenotype, which cannot always be taken into account, and the utilization of different models and approaches [13,14,15,16,17,18,19,20,21,22]. At the same time, other studies have identified the same candidate genes associated with trait variability in different animal species [23]. The idea of preliminary identification and selection of significant SNP markers for inclusion in calculating genomic estimates served as the basis for developing the weighted ssGBLUP procedure [11,24]. In addition, B. Fragomeni et al. (2017) suggested that the proportion of significant markers in the total SNP number can serve as an indirect indicator of the trait polygenicity (“complexity”). They also mention the relatively low efficiency of significant markers identified by any known methods concerning complex quantitative traits [24]. Therefore, the idea of redistributing the “weight” (contribution) of each marker to the individual genetic value assessment while maintaining a standard set of SNP markers on microarray chips (preferably of high density) looks the most promising [25]. Many studies based on real and simulated data compared the effectiveness of using reduced sets of SNP markers through methods such as pre-selection based on GWAS, data from dense and medium-density chips, and whole-genome information. However, improvement in the accuracy of the estimates for the limited marker sets was not always observed (except when the causal genes (QTL) are known), and an increase in the distortion of the results due to genotyping errors is also mentioned by some authors [6,8,26,27,28,29]. The fact is that SNPs explain a part of the complex trait heritability, and each of them individually has an infinitely small effect size. Moreover, the main issue is not the number of identified loci influencing the appearance of the trait but rather how the identified loci cumulatively explain the appearance of the trait [30] and whether it can be effectively used for individual prediction, for example, when conducting a genomic evaluation. The assessment of SNPs affects a significant influence on the particular trait phenotype manifestation variability, and additionally, the values of the SNP markers affect themselves. This depends dramatically on the reference group parameters used for calculation, in particular, the allele frequencies distribution, the linkage disequilibrium extent, the genetic structure, the effective size of the reference population, as well as the polygenicity of the trait itself (the presence of «major» QTLs with significant effects) [7]. Due to this, the competent and reasonable formation of a sufficient size reference group genotyped on high-density chips plays an essential role in forming the individual genomic assessment [6].

Therefore, our study aimed to assess the impact of the SNP genotypes’ data amount on individual genomic estimate accuracy based on the ssGBLUP and wssGBLUP methods. The validity of applying the weighting procedure to improve the accuracy of genomic predictions for pigs’ meat, fattening, and reproduction traits were considered.

## 2. Materials and Methods

### 2.1. Phenotypes

The phenotypic features of meat, fattening and reproductive traits of Large White pigs were used. Animals born between 2015 and 2020 were included in the dataset. The following characteristics of meat and fattening productivity were evaluated: fat thickness over 6–7 ribs (BF1, mm), muscle depth (MD, mm) and precocity up to 100 kg (age, days). In addition, according to the results of the first farrowing, the number of all-born piglets (TNB) and the number of live-born piglets (NBA) were taken into account. Descriptive statistics of the complete data set are given in Table 1.

To assess the genomic data amount effect on the accuracy of predictive values for several traits, we formed six reference groups (groups of genotyped animals) and calculated them according to the scenarios presented in Table 2.

The groups were selected in such a way as to simulate a situation where genotyping was carried out on the part of the livestock in the estimated population at three stages, with a cumulative effect. An analysis of meat and fattening qualities was carried out on a dataset that included 62,927 records of in vivo measurements of backfat thickness, muscle depth and precocity in gilts and boars when they reached 100 kg live weight. Studies of reproductive traits were carried out on a dataset, including results of the first farrowing (16,070 observations). Accordingly, the reference groups of genotyped individuals according to the meat and reproduction traits were different but formed according to a single algorithm. Thus, the first group for meat and fattening qualities included 530 pigs (boars, sows, piglets) from the 2010–2015 year of birth, and the second group included 1178 individuals, including the first group (boars, sows, piglets) born in 2010–2017. The third group consisted of animals from the second group, to which 315 more heads were added, for a total of 1493 individuals born from 2010–2021. According to the reproduction traits, only genomic data of sows were used in the analysis. Therefore, the following values characterized the groups: the fourth included 396 individuals (sows born from 2010–2015), the fifth included 870 animals (sows born from 2010–2017), and the sixth included 1228 sows born from 2010–2020 (Table 3). Based on the groups’ genomic data, the values of the genomic scores of all individuals in the total analyzed data sets were determined.

The most complete (numerous) reference groups of individuals 3 and 6 were characterized by the following average indicators and standard deviations of phenotypic values (mean (SD)): group 3, BF1—14.61 (3.13), MD—57.95 (5.96), age—156.1 (9.78); group 6, TNB—13.48 (3.87), NBA—12.76 (3.75). The phenotype’s limiting values (ranges) of the reference groups did not differ from those of the general population. A set of partial data was formed for validation by deleting records of validated animal phenotypes. The relationships between the validated animals and their descendants were reset in the pedigree. The group of validated animals for meat and fattening traits included 200 individuals: 79 boars (mean rel = 0.954) and 121 sows (mean rel = 0.849). The validated animals for reproduction traits group included 165 sows (mean rel = 0.572).

### 2.2. Genotypes

We utilized the GGP Porcine HD hybridization chips (Illumina/Neogen, Lincoln, NE, USA) to genotype boars and sows used in this study to form reference groups. The GGP Porcine HD hybridization chip contains 70,000 SNPs with a mean intermarker interval of approximately 42 kb and bears 20 major causative mutations. These SNPs are identified within and between all major pig breeds. Genome-wide genotyping data were presented as 1483 individual genotypes exported from the GenomeStudio 2 program (Illumina Inc., San Diego, CA, USA).

We performed data quality control (QC) by using Plink 1.9 [31] to ensure the accurate identification of SNPs and for the correct interpretation of genetic association results. Individuals and SNPs that did not pass the following thresholds were removed: SNPs with accuracy (GC Score, GT Score) less than 0.2, animal records with more than 10% genotype absence, SNP markers absent in more than 10% of genotyped animals, SNP markers with a minor allele frequency of less than 5%; SNP markers deviating from the Hardy–Weinberg equilibrium with a threshold value of 10^−6^; and animal records with Mendelian errors of allele inheritance (the presence of such errors was regarded as errors in recording animal pedigrees). Afterward, the QC genomic data included 50,740 SNPs of 1483 genotyped animals.

### 2.3. Statistical Analysis and Assessment

Descriptive statistics calculations were performed in the program STATISTICA 10, and animal pedigrees were analyzed by the CFC software package [32]. Primary processing and transformation of genomic data were carried out using the local programs using integrated development environment (IDE) RStudio for the R language. SNeP v1.1 was used to compute effective population sizes [33], and the BLUPF90 software package

Reference [34] was used to obtain individual breeding value estimates (EBV, GEBVss, GEBVwss). The genetic population parameters (heritability, genetic variance and covariance) were estimated based on the restricted maximum likelihood method in the REMLF90 program [34,35].

The mixed linear model on the BLUP AM method is:

*y* = *Xb* + *Za* + *e*,

where *y* represents the vector of phenotype observations on the trait (TNB, NBA, BF1, MD and Age); *b* represents vector of fixed effects («farm-year—contemporary group»; «sex of the animal»; «weight of the animal»); *a* represents vector of random animal effect; *e* represents the vector of the random residual effect; and *X* and *Z* represent the incidence matrices.

The significance of the fixed effects was assessed based on the exact Fisher criterion. To optimize the set of random effects, the Akaike criterion was used [36]. The genomic relationship matrix *G* was obtained using the linear method proposed by Van Raden [37]:

$$G=\frac{M^{\prime }M}{2\sum_{i=1}^{m}p_{i}\left(1-p_{i}\right)}$$

where *m* is the number of SNPs, *p_i_* is the frequency of the minor allele of SNP *I*, and *M* is the centered genotype matrix.

Fixed and random effects in the ssGBLUP and wssGBLUP models are the same as in the BLUP AM model. The relationship matrix (*A*^−1^) is replaced by (*H*^−1^).

$$H^{-1}=A^{-1}+\left[\begin{array}{cc} 0 & 0\\ 0 & \tau {\left(\alpha G+\beta A_{22}\right)}^{-1}-\omega A_{22} \end{array}\right]$$

where *A* is the relationship matrix, *A*_22_^−1^ is the inverse relationship matrix for genotyped animals, *G* is the genomic relationship matrix, and the coefficients *α* = 0.95, *β* = 0.05, τ = ω = 1 (by default) [38].

The EBV reliability (GEBV) was calculated as follows:

$$Rel=1-\frac{PEV}{varA}$$

where *PEV* is the estimated error variance, and *varA* is the additive genetic variance.

In the wssGBLUP approach (weighted ssGBLUP) genomic matrix (*G*) is replaced by a weighted G* matrix, which is constructed as follows:

$$G^{*}=\frac{M^{\prime }DM}{2\sum_{i=1}^{m}p_{i}\left(1-p_{i}\right)}$$

In the wssGBLUP analysis, the *D* matrix is a diagonal matrix of the SNP weights [11]. SNP weights were obtained by solving the inverse GEBV equation using POSTGSF90 software [36] based on the non-linear method for calculating SNP weights (by Van Raden [37]):

$$d_{ii}=1.125^{\frac{\left|\hat{a_{i}}\right|}{sd\left(\hat{a}\right)}-2}$$

The wssGBLUP approach is based on an iterative algorithm with the following steps: (1) perform ssGBLUP with matrix G* (at iteration 1, all SNP weights in matrix D are equal to 1 and are equivalent to ssGBLUP), (2) estimate the effects of SNP values of genomic scores in the previous step, (3) evaluate the effect of each SNP, (4) normalize the SNP effect vector to obtain SNP weights (the normalization process ensures that the sum of the SNP variances remains constant), (5) use the SNP weights to construct a new D-matrix, and (6) move to step (1).

## 3. Results

The pig breeding value estimates and their reliability were calculated according to three meat and fattening traits and two reproduction traits. Genomic scores (GEBV) were obtained using all three compared reference groups (Scenarios 1–3) for genotyped animals of the smallest groups: the first and fourth. A comparison of the average reliability values of the GEBV estimates indicates that accuracy grows for each analyzed method with increasing the reference group size from +4.0 to +9.9% points and from +0.0 to +2.4% points for the ssGBLUP and wssGBLUP methods, respectively. Thus, the accumulation of genomic data for the reference group gives the most significant increase in accuracy when using the ssGBLUP method. In contrast, the reference group size enlargement leads to a relatively smaller increase in the prediction accuracy for the wssGBLUP method.

In the previous article, we discussed the increase in the accuracy of breeding value estimates by including genomic data on the characteristics of meat and fattening productivity and reproduction traits in pigs [39]. However, in this study, we decided to evaluate the validity of the procedure application for individual SNP marker effect weighting and compared the obtained estimates’ reliability with traditional BLUP AM (Scenario 4) and ssGBLUP (Table 4).

We note that the wssGBLUP method has an advantage over ssGBLUP in estimated reliability. For example, when using a small reference group (Scenario 1), the difference in the accuracy of ssGBLUP over BLUP AM is from −1.9 to + 7.3 percent points, while for wssGBLUP, the change in accuracy varies from +18.2 to +87.3 percent points. Furthermore, the superiority of the second method is also maintained for the largest group of genotyped animals (Scenario 3): from +4.7 to +15.9 percent points for ssGBLUP and from +21.1 to +90.5 percent points for wssGBLUP. The application of the wssGBLUP method using the genomic data for a reference group of 500 animals (Scenario 1) at first glance already makes it possible to obtain highly reliable predictive values of the pig breeding values in terms of meat fattening productivity and reproduction indicators.

The correlation coefficients between animal estimates of the first group (530 animals) GEBVwss and GEBVss based on meat and fattening productivity, obtained using three reference groups, were close to 1 (from 0.950 to 0.994 for GEBVss and from 0.970 to 0.995 for GEBVwss). In our opinion, this indicates the high reliability of both methods concerning highly inherited traits. On the other hand, for reproduction traits, the correlation coefficients between estimates were characterized by lower Pearson correlation values: from +0.823 to +0.980 for GEBVss and from +0.878 to +0.981 GEBVwss (results not shown in the table).

The validation of genomic estimates using ssGBLUP and wssGBLUP methods was carried out by comparing genomic predictions based on a partial data set (PD) (Scenario 5) with the most accurate genomic estimates based on complete information about animals, whole data set (WD) (Scenario 3) (Table 5).

A preliminary evaluation of young animals was performed based on the pedigree and was a prediction based on the parent average (PA). In our study, the predictive indicators of a validated group of individuals on a partial data set (PA_PD_, GEBV_ssPD_, GEBV_wssPD_) were compared with their most accurate breeding value estimates (GEBV_wssWD_) on the whole data set.

A genomic estimation method validation was carried out on genomic predictions correlated with highly reliable estimates for each method (Scenario 5). Of note is the fact that for traits with relatively higher heritability (meat and fattening qualities), the relationship of the predicted values with the final estimates was the lowest for PA_PD_, with the exception of the correlation r(PA_PD_, GEBV_ssWD_). Estimates based on weighted values are either close to or slightly inferior to the GEBV_ssPD_ estimates. The final estimates (EBV_WD_, GEBV_ssWD_, GEBV_wssWD_) have the highest correlation with predictions calculated using the same method. However, highly reliable estimates based on BLUP are more closely interrelated with genomic predictions. The final weighted estimates correlate almost at the same level with the predictions of GEBV_ssPD_ and GEBV_wssPD_. For traits with low heritability (TNB, NBA), GEBV_wssWD_ scores are also inferior to GEBV_ssWD_. In the case of traditional BLUP, the greatest relationship is characterized by PA_PD_. It follows from the results that genomic methods are most justified in predicting the true breeding value of individuals for traits with relatively high heritability. For low-inherited traits, the ssGBLUP method has an advantage over wssGBLUP.

This is also confirmed by the analysis of the inflation of the final estimates for preliminary predictions (EBV_WD_, GEBV_ssWD_, GEBV_wssWD_ on PA_PD_, GEBV_ssPD_, GEBV_wssPD_). The level of the regression coefficient (b), when predictions and final estimates were based on the same method, varied. For the BLUP method, it ranged from 0.64 to 0.72 (R^2^ from 0.11 to 0.15) and 1.06 (R^2^ from 0.59 to 0.62); for the ssGBLUP method, it ranged from 0.78 to 0.87 (R^2^ from 0.26 to 0.34) and from 0.99 to 1.01 (R^2^ from 0.77 to 0.77); and for the wssGBLUP method, it ranged from 0.57 to 0.78 (R^2^ from 0.21 to 0.32) and from 0.76 to 0.77 (R^2^ = 0.51) for meat and fattening traits and reproduction traits, respectively. In the case when different methods obtained the prediction and final estimates, the best values of inflation (close to one) and the coefficient of determination were identified for GEBV_ssPD_ (from 0.70 to 0.88) (R^2^ from 0.20 to 0.32) for meat and growth traits, from 0.69 to 1.27 (R^2^ from 0.47 to 0.48) for reproduction traits), the worst values of b for meat and fattening traits had PA_PD_, and for reproduction traits—GEBV_wssPD_ (the data is given in the Supplementary Table S1).

In our opinion, such a significant increase in the reliability of GEBVwss estimates compared to GEBVss is associated with a decrease in the residual variance due to the use of more optimal contributions of SNP markers when using the weighting procedure for the effect of each SNP marker (with the constant genetic variance). However, the validation of genomic methods (ssGBLUP and wssGBLUP), a test of the accuracy of genomic estimates [10], indicates a relatively higher predictive ability of the analyzed methods of the genomic assessment of complex polygenic traits in comparison with the assessment by pedigree, it reveals a slight improvement in the prognosis due to the use of the weighting procedure. This is due to several factors: the absolute effect values of individual SNP markers are very small, and there are no main effects for the analyzed traits. Thus, with a given limited amount of genomic data (approximately 1.5 thousand samples), a change in the set of phenotypic data (the phenotypes of the validated group were excluded from the calculation) leads to a significant change in these effect estimates and, accordingly, to a redistribution of each marker weight, which is observed during validation. For a given amount of genomic data, the wssGBLUP method is associated with the risk of obtaining biased results if there is a significant change in the reference group based on which the marker weights were estimated (which will be shown below).

We analyzed the levels of the EBV relationship for BLUP AM (Scenario 4) in individuals of the first and fourth groups, obtained for three methods using genomic data from different reference groups (Scenarios 1–3) (Table 6).

As can be seen from the results, an increase in the size of the reference group for both methods reduces the relationship level between EBV and GEBV scores, which is most pronounced for the GEBV_wss_ estimates, apparently due to the adjustment of marker effects.

It should be noted that the level of correlation between the genomic estimates themselves, obtained by the two compared methods, is very high (for meat and fattening traits r_(GEBVss, GEBVwss)_ varies from +0.887 to +0.999 depending on the group; for reproduction traits it varies from +0.737 to +0.868), and from the point of view of pigs practical selection, these differences in results are not so significant. However, the change in the correlation coefficients indicates a stable decrease in the relationship between genomic scores and EBV for meat and fattening traits with the accumulation of genomic information, while changes in reproductive qualities are less predictable.

We analyzed the values of the SNP marker weights based on the contribution of each marker effect to the final breeding value assessment, as well as the part of the total genetic variance described by each marker and the *p*-values characterizing the significance of the marker effect contribution into the trait variability (Table 7).

The correlation coefficient analysis of the variance proportions explained by SNP markers indicates a redistribution of these values for groups that differ significantly in size (1 and 3, 4 and 6), which was also confirmed by the relatively low consistency of the SNP markers’ weight values. Each marker’s contribution varies depending on the reference group examined (Scenarios 1–3). A similar trend was observed for the *p*-values, confirming the more remarkable similarity of the significant marker sets identified in groups 2 and 3. The SNP marker allele frequencies analysis revealed a slight change in the compared reference groups. The correlation of allele frequencies between different groups was as follows: r_(gr1,gr2)_ = +0.929, r_(gr1, gr3)_ = +0.901, and r_(gr2, gr3)_ = +0.959 for meat and fattening traits, and r_(gr1,gr2)_ = +0.936, r_(gr1, gr3)_ = +0.904, and r_(gr2, gr3)_ = +0.959 for reproduction traits.

**Figure 1:**
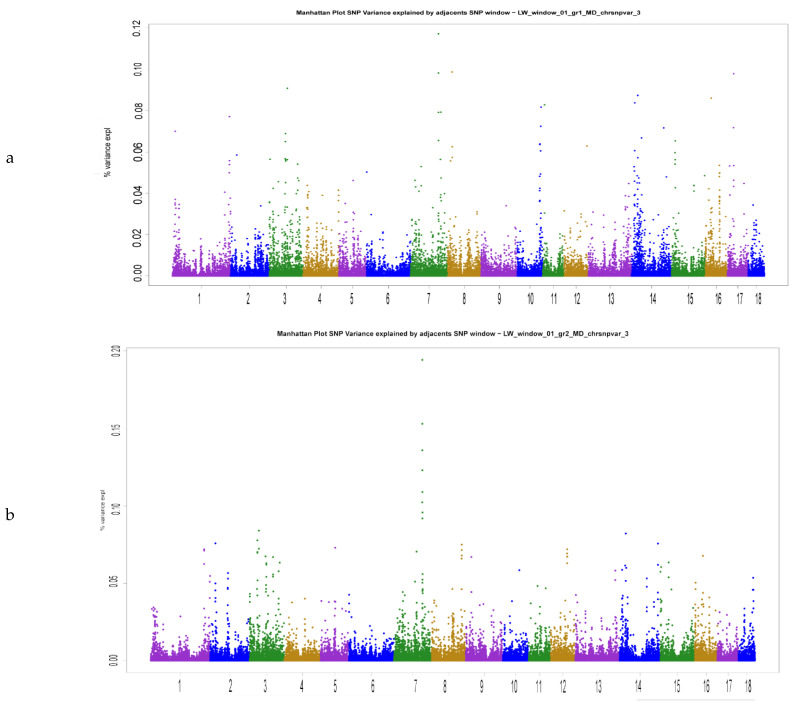

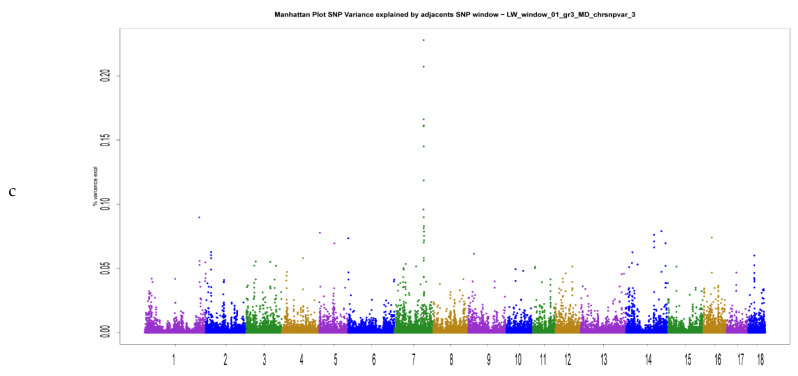
Manhattan plots of muscle depth (MD) variance explained by SNP markers based on Scenarios 1–3 (**a**—Scenario 1, **b**—Scenario 2, **c**—Scenario 3).

**Figure 2:**
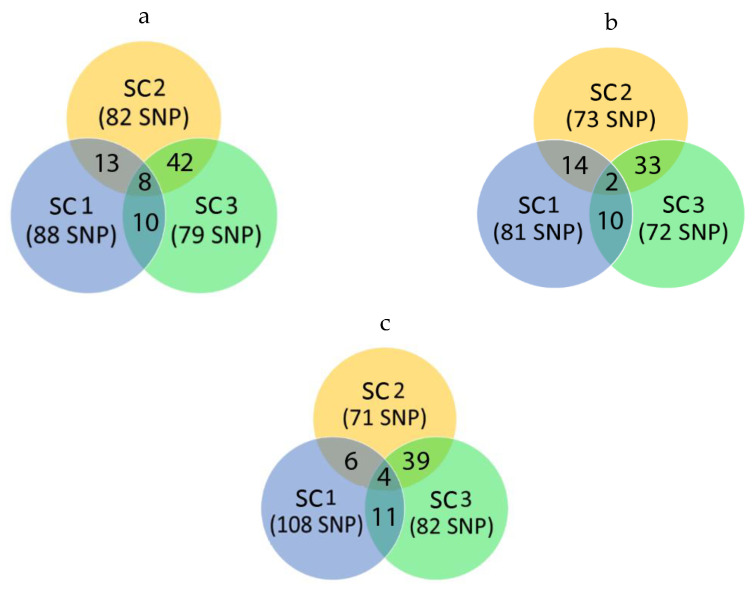

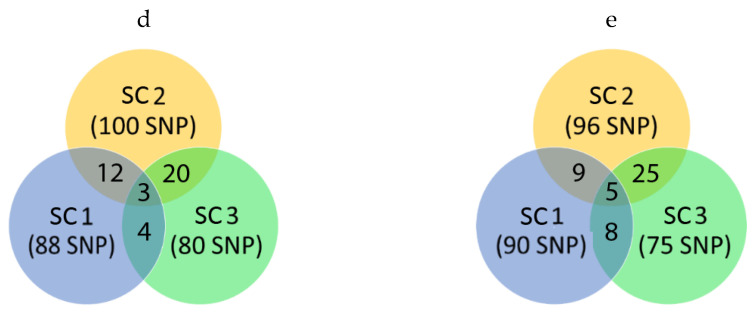
Trait/Venn diagrams showing common SNP in different groups. Values in brackets indicate the total number of SNP markers which explain 5% of a trait variability based on the respective reference group. Set intersections indicate the number of SNP markers that explain 5% of the trait variability common for calculations based on Scenarios 1–3 (SC1—Scenario 1, SC2—Scenario 2, SC—Scenario 3); **a**—for backfat thickness over 6–7 ribs (BF1), **b**—for muscle depth (MD), **c**—for days to 100 kg (Age), **d**—for number of all piglets born at the first farrowing (TNB), **e**—for number of piglets born alive at the first farrowing (NBA).

The power of the method to take into account the individual SNP marker weight as a predictor of breeding value becomes significantly lower in situations when the true QTLs of the greatest influence on the trait variability are unknown and the traits themselves are polygenic. The traits analyzed in the study are characterized by very low proportions of variance described by individual SNP markers (Figure 1). Thus, about 5% of the genetic variance of the BF1 trait is explained by 88 SNP markers when Scenario 1 using. For all analyzed traits, the number of markers explaining 5% of genetic variability varied from 71 to 108, and the number of such SNPs varied depending on the reference group size (79–88 for BF1, 72–81 for MD, 71–108 for age) (Figure 2).

It is noteworthy that the results obtained for the genetic variance distribution over the entire set of SNP markers for the analyzed traits have the most remarkable similarity between the groups Scenario 2 and 3. A reference group size of about 1000 (Scenario 2) and about 1500 heads (Scenario 3) gives more consistent results for evaluation based on the wssGBLUP method. Thus, applying this procedure using a reference group of 500 heads can lead to somewhat distorted GEBV results.

## 4. Discussion

Causal QTN information can be included in the ssGBLUP procedure using a weighted genomic relatedness matrix. For example, a study by [24] performed on simulated data showed that the inclusion of realistic weights for causal QTNs allows for genomic estimates of higher accuracy. Moreover, it was assumed that the pre-selection of SNP markers (as a tool for optimizing the model prediction) should increase the accuracy of genomic estimates. However, studies [29] for the Japanese flounder (*Paralichthys olivaceus*) based on disease resistance showed that a significant increase in the prediction reliability was not observed with the preliminary selection of SNPs based on both SNP-GWAS (by *p*-value) and ssGWAS (by a fraction of the marker explained variance) [29]. In this connection, the authors conclude that the preliminary marker selection does not significantly increase the accuracy of the estimates while analyzing low heritable traits. In our study, the results also indicate a rather significant redistribution of the genetic variance proportions described by individual SNP markers of a trait, which does not allow for a preliminary marker selection for genomic prediction based on the estimated traits in pigs.

A comparison of the genomic estimate accuracy obtained using wssGBLUP with traditional BLUP estimates was carried out by [40] for Holstein cows based on calving interval. The gain in GEBV accuracy with wssGBLUP was +5.4 to +5.7 (first-calf heifers) and +9.4 to +9.7 (multi-calving cows) percent points compared to pedigree-based BLUP accuracy [40]. Lourenco et al. [25] also compared the genomic prediction accuracy for dairy productivity traits in cows obtained using several methods (Bayesian regression, genomic BLUP, single-step GBLUP and weighted single-step GBLUP). The authors found the superiority of the ssGBLUP method for populations with relatively few genotyped animals. However, it was also noted that weighted ssGBLUP has the potential to improve the accuracy of the estimates [25]. Lourenco et al. showed on simulated data that wssGBLUP is more efficient when the genotyping population is small and a small number of QTLs affect the trait. When using medium-size genotyped populations, ssGBLUP can be applied without losing accuracy compared to wssGBLUP [41].

The most accurate marker weights in the study by Atashi et al. [40] were obtained at the second iteration of the wssGBLUP procedure. Similar results for SNP marker weight accuracy assessment at the second iteration were obtained in the study by Mehrban et al. (2021) using a non-linear calculation method for meat traits and carcass quality in beef cattle. Hanwoo [42], as well as Teissier M et al. [43,44], assessed for various traits in dairy goats using the quadratic SNP weighting procedure and windows of 40 SNPs. Our study found the highest estimate accuracy at the third iteration for all analyzed traits, similar to the study by Zhang et al. (2016) [45] on simulated data. The effectiveness of the weighted SNP effects was compared on dairy goats [43] for milk protein content. As a result, the authors show that the weighted ssGBLUP is more accurate than ssGBLUP (+8 to +12 percent points). The same authors [44] found that the wssGBLUP method gives a slight advantage over ssGBLUP and only for traits with identified QTLs when studying milk productivity traits, udder quality, and the number of somatic cells in goat milk [43,44]. Our research results confirm the increase in genomic estimate reliability when using wssGBLUP compared to ssGBLUP and BLUP AM. Moreover, the increase in the reliability of GEBV_wss_ relative to EBV ranges from +18.2 to +90.5 percent points. Mehrban et al. (2021) showed a non-uniform improvement in the accuracy of beef trait weighted scores in beef cattle compared to ssGEBV [42]. Although accuracy gains ranged from 5 to 13 percentage points (when using individual SNPs), for BF (fat thickness) and MS (marbling score), significant accuracy gains between ssGBLUP and wssGBLUP were not observed.

Embedding genomic assessment into the overall livestock breeding value assessment system implies the presence of a reference population (group) of genotyped animals of sufficient size with optimal parameters to obtain the most reliable genomic prediction results. However, the issue of determining these parameters and volume still cannot be resolved unambiguously. Alvarenga et al. (2020) [46] compared the ssGBLUP and wssGBLUP methods based on different training populations (only purebred individuals and the combined population—purebred plus crossbreeds). As a result, it was found that the ssGBLUP method gave the highest accuracy and the smallest GEBV bias. However, the authors noted the stratification of the genotyped population (purebred and crossbreeds) [46]. In our study, there was no stratification in the compared groups; therefore, the advantage of the wssGBLUP method was observed.

Ardestani et al. (2021) [3] proved the superiority of genomic methods for predicting breeding value over the traditional BLUP procedure in Duroc, Landrace and Yorkshire pigs for meat traits. The greatest estimate reliability for fat thickness, muscle depth and average daily gain was achieved by ssGBLUP and BayesCp. However, the increase in reliability was different for each breed studied. According to the authors, this occurrence can be caused by differences in the reference group’s volume, the amount of data available for evaluation, and the differences in the breeding selection goals [3].

The accuracy of genomic estimates in the study by Grossi et al. (2018) [27] on pigs of three breeds (Duroc, Landrace and Yorkshire), calculated as the Pearson’s correlation of deregressed breeding value estimates with genomic predictions for fat thickness, muscle depth, and number of piglets born ranged from 0.39 to 0.55 (by BF1), from 0.40 to 0.68 (by MD), and from 0.47 to 0.58 (by TNB) with a decrease in these indicators with a decrease in the number of included SNP markers (by the development of low-density panels), and slightly exceeded the pedigree-based prediction (for BF1 accuracy, this value ranged from 0.40 to 0.54; for MD, it ranged from 0.52 to 0.61; for TNB, it ranged from 0.26 to 0.64) [27]. In our study, validation revealed a similar level of correlation between wssGBLUP and ssGBLUP genomic predictions (Scenario 5) with highly accurate final estimates of GEBV_wssWD_ (Scenario 3): for BF1, values ranged from 0.51 to 0.56; for MD, values ranged from 0.45 to 0.50; for age, values ranged from 0.43 to 0.48; for TNB, values ranged from 0.69 to 0.71; and for NBA, values ranged from 0.69 to 0.72, which exceeds the accuracy of predictions based on the pedigree using the BLUP AM method (the correlation of PA with GEBV_wssWD_ was 0.36 for BF1, 0.34 for MD, 0.39 for age, 0.62 for TNB and 0.57 for NBA).

It should be noted that the genomic assessment procedure (based on the ssGBLUP and wssGBLUP methods) can significantly improve the preliminary prediction accuracy of the individual genetic values within a particular population. An increase in the reference group size provides more stable individual marker weight values, and the proportion of genetic variance explained by each of them, especially when the reference group is closely related to the assessed population [23]. Since the strength of the LD between SNP markers and QTL may decay over time due to the impact of genetic forces, genomic predictions calculated based on the reference group may lose accuracy. In this regard, constant retraining of the prediction model, reassessment of SNP effects and constant replenishment of the phenotypic data set are required [7]. An additional increase in the genomic prediction reliability can be achieved by using SNP marker weights, which should be calculated separately for each trait [25].

## 5. Conclusions

Using the ssGBLUP and wssGBLUP methods makes it possible to assess pigs’ genomic breeding values based on the main breeding traits (meat and fattening qualities and reproduction indicators of the first farrowing) with high precision. Using the weighting procedure to evaluate SNP markers effects significantly improves the accuracy of the genomic predictions. The wssGBLUP implementation is reasoned for genotyped reference groups of as few as 1000 individuals (meat and fattening qualities) or 1500 animals (reproduction indicators). Further replenishment of the reference group involves the mandatory recalculation of the SNP markers weights for genomic assessment of polygenic traits influenced by a large number of QTLs, such as meat, fattening and reproduction qualities in pigs.

## Figures and Tables

**Table 1:** The descriptive statistics of the whole dataset.

Trait	Mean	SD	Range	h^2^	N
BF1	16.13	3.63	7.0 to 34.0	0.428	62,927
MD	59.52	7.18	40.0 to 99.0	0.195	
Age	154.4	9.17	109 to 205	0.323	
TNB	15.4	4.14	1 to 29	0.119	16,070
NBA	14.2	3.95	0 to 28	0.112	

**Table 2:** Calculation scenarios.

Scenario	Methods	Phenotypes and Pedigree Data	Genomic Data	Results
Scenario 1	ssGBLUP wssGBLUP	WD	RG1 (RG4)	GEBV_ss1/4_ GEBV_wss1/4_
Scenario 2	ssGBLUP wssGBLUP	WD	RG2 (RG5)	GEBV_ss2/5_ GEBV_wss2/5_
Scenario 3	ssGBLUP wssGBLUP	WD	RG3 (RG6)	GEBV_ss3/6_ GEBV_wss3/6_
Scenario 4	BLUP AM	WD	-	EBV_WD_
Scenario 5 (validation)	BLUP AM		-	PA_PD_
	ssGBLUP	PD	RG3 (RG6)	GEBV_ssPD_
	wssGBLUP		RG3 (RG6)	GEBV_wssPD_

**Table 3:** Reference groups description.

Parameter	Meat and Fattening Traits		
	Group 1	Group 2	Group 3
N	530	1178	1493
Ne	85	90	96
Reproduction traits			
	Group 4	Group 5	Group 6
N	396	870	1228
Ne	96	107	107

N: number of animals in groups; Ne: effective population size.

**Table 4:** Average animal estimate reliability of the first and fourth reference groups (530 and 396 animals), depending on the method and amount of genomic data.

Trait	BLUP AM	ssGBLUP			wssGBLUP		
Meat and fattening traits							
	Scenario 4	Scenario 1	Scenario 2	Scenario 3	Scenario 1	Scenario 2	Scenario 3
BF1	0.723	0.716	0.757	0.767	0.919	0.931	0.933
MD	0.597	0.606	0.654	0.666	0.931	0.942	0.944
Age	0.679	0.675	0.718	0.728	0.983	0.986	0.986
Reproduction traits							
NBA	0.438	0.465	0.484	0.499	0.803	0.803	0.813
TNB	0.441	0.473	0.492	0.511	0.826	0.828	0.840

BF1: backfat thickness over 6–7 ribs, MD: muscle depth, Age: days to 100 kg, TNB: number of all piglets born at the first farrowing, NBA: number of piglets born alive at the first farrowing.

**Table 5:** Correlation of the weighted final genomic estimates (GEBVwssWD) of the validated group (200 animals) with their parent’s average predictions (PA), genomic predictions based on pedigree and genome data using the ssGBLUP (GEBV_ssPD_) and wssGBLUP (GEBV_wssPD_) methods (Scenario 5) *.

Correlation between		Trait				
		BF1	MD	Age	TNB	NBA
EBV_WD_	PA_PD_	0.358	0.336	0.387	0.787	0.767
	GEBV_ssPD_	0.509	0.500	0.452	0.697	0.690
	GEBV_wssPD_	0.505	0.436	0.426	0.618	0.594
GEBV_ssWD_	PA_PD_	0.624	0.506	0.699	0.751	0.721
	GEBV_ssPD_	0.993	0.942	0.969	0.875	0.878
	GEBV_wssPD_	0.569	0.520	0.488	0.799	0.791
GEBV_wssWD_	PA_PD_	0.356	0.278	0.373	0.615	0.574
	GEBV_ssPD_	0.565	0.498	0.479	0.686	0.693
	GEBV_wssPD_	0.562	0.457	0.466	0.713	0.716

* All correlation coefficients are significant with *p*-values < 0.001. BF1: backfat thickness over 6–7 ribs, MD: muscle depth, Age: days to 100 kg, TNB: number of all piglets born at the first farrowing, NBA: number of piglets born alive at the first farrowing.

**Table 6:** Correlation of EBV/GEBV of individuals of the first and fourth reference groups (530 and 396 heads) obtained by compared methods *.

EBV by Trait	GEBVss			GEBVwss		
Meat and fattening traits						
Scenario 4	Scenario 1	Scenario 2	Scenario 3	Scenario 1	Scenario 2	Scenario 3
EBV(BF1)	0.974	0.950	0.945	0.977	0.956	0.951
EBV(MD)	0.957	0.927	0.910	0.926	0.918	0.911
EBV(Age)	0.961	0.937	0.931	0.965	0.948	0.944
Reproduction traits						
EBV(NBA)	0.944	0.781	0.823	0.792	0.843	0.841
EBV(TNB)	0.944	0.769	0.808	0.775	0.840	0.846

* All correlation coefficients are significant with *p*-values < 0.001. EBV(BF1): estimated breeding value on BLUP AM by backfat thickness over 6–7 ribs, EBV(MD): estimated breeding value on BLUP AM by muscle depth, EBV(Age): estimated breeding value on BLUP AM by days to 100 kg, EBV(TNB): estimated breeding value on BLUP AM by the number of all piglets born at the first farrowing, EBV(NBA): estimated breeding value on BLUP AM by the number of piglets born alive at the first farrowing.

**Table 7:** Correlation of parameters of compared reference groups.

Trait/Group		Part of Variance Explained by Each SNP Marker		*p*-Value *		SNP Marker «Weight»	
Meat and fattening traits							
		Scenario 1	Scenario 2	Scenario 1	Scenario 2	Scenario 1	Scenario 2
BF1	Scenario 2	0.429	-	0.382	-	0.408	-
	Scenario 3	0.352	0.850	0.314	0.834	0.352	0.839
MD	Scenario 2	0.511	-	0.454	-	0.479	-
	Scenario 3	0.435	0.816	0.353	0.790	0.390	0.796
Age	Scenario 2	0.394	-	0.366	-	0.404	-
	Scenario 3	0.335	0.822	0.291	0.809	0.330	0.811
Reproduction traits							
NBA	Scenario 2	0.424	-	0.395	-	0.436	-
	Scenario 3	0.318	0.720	0.277	0.714	0.331	0.732
TNB	Scenario 2	0.420	-	0.384	-	0.423	-
	Scenario 3	0.294	0.725	0.263	0.716	0.313	0.733

* All correlation coefficients are significant with *p*-values < 0.001. BF1: backfat thickness over 6–7 ribs, MD: muscle depth, Age: days to 100 kg, TNB: number of all piglets born at the first farrowing, NBA: number of piglets born alive at the first farrowing.

## Data Availability

The dataset (phenotype and genotype information) of the Large White pigs used and analysed during the current study are owned by LLC “TOPGEN” and were provided via a signed data access agreement, which does not allow for data sharing.

## Supplementary Materials

The following supporting information can be downloaded at: https://www.mdpi.com/article/10.3390/ani12131693/s1, Table S1: Regression coefficients (b) of final estimates (EBV_WD_, GEBV_ssWD_, GEBV_wssWD_) on predictions (PA_PD_, GEBV_ssPD_, GEBV_wssPD_) of validated animals.
