# Supplementary material for: Weighted Single-Step Genomic Best Linear Unbiased Prediction Method Application for Assessing Pigs on Meat Productivity and Reproduction Traits
Source: Animals (Basel). 2022 Jun 30;12(13):1693. doi: 10.3390/ani12131693 (PMC9264777; doi:10.3390/ani12131693)
Supplement: Supplementary file 1 [file animals-12-01693-s001.zip › animals-1666279-supplementary.pdf]

Supplementary Table S1. Regression coefficients (b) of final estimates ($EBV_{WD}$, $GEBV_{ssWD}$, $GEBV_{wssWD}$) on predictions ($PA_{PD}$, $GEBV_{ssPD}$, $GEBV_{wssPD}$,) of validated animals*

| Pair of estimates / Trait | BF1 | MD | Age | TNB | NBA |
|---|---|---|---|---|---|
| b($EBV_{WD}$, $PA_{PD}$) | 0.668 ($R^2$ = 0.128) | 0.720 ($R^2$ = 0.113) | 0.637 ($R^2$ = 0.150) | 1.060 ($R^2$ = 0.619) | 1.061 ($R^2$ = 0.589) |
| b($EBV_{WD}$, $GEBV_{ssPD}$) | 0.747 ($R^2$ = 0.259) | 0.762 ($R^2$ = 0.250) | 0.696 ($R^2$ = 0.204) | 0.691 ($R^2$ = 0.486) | 0.688 ($R^2$ = 0.476) |
| b($EBV_{WD}$, $GEBV_{wssPD}$) | 0.687 ($R^2$ = 0.255) | 0.467 ($R^2$ = 0.190) | 0.509 ($R^2$ = 0.181) | 0.366 ($R^2$ = 0.382) | 0.346 ($R^2$ = 0.353) |
| b($GEBV_{ssWD}$, $PA_{PD}$) | 0.669 ($R^2$ = 0.131) | 0.661 ($R^2$ = 0.099) | 0.637 ($R^2$ = 0.154) | 1.151 ($R^2$ = 0.564) | 1.154 ($R^2$ = 0.519) |
| b($GEBV_{ssWD}$, $GEBV_{ssPD}$) | 0.835 ($R^2$ = 0.328) | 0.867 ($R^2$ = 0.335) | 0.777 ($R^2$ = 0.261) | 0.986 ($R^2$ = 0.765) | 1.014 ($R^2$ = 0.772) |
| b($GEBV_{ssWD}$, $GEBV_{wssPD}$) | 0.770 ($R^2$ = 0.324) | 0.545 ($R^2$ = 0.269) | 0.576 ($R^2$ = 0.238) | 0.538 ($R^2$ = 0.638) | 0.534 ($R^2$ = 0.626) |
| b($GEBV_{wssWD}$, $PA_{PD}$) | 0.673 ($R^2$ = 0.127) | 0.689 ($R^2$ = 0.077) | 0.675 ($R^2$ = 0.139) | 1.490 ($R^2$ = 0.378) | 1.459 ($R^2$ = 0.329) |
| b($GEBV_{wssWD}$, $GEBV_{ssPD}$) | 0.841 ($R^2$ = 0.319) | 0.880 ($R^2$ = 0.248) | 0.813 ($R^2$ = 0.230) | 1.222 ($R^2$ = 0.470) | 1.270 ($R^2$ = 0.480) |
| b($GEBV_{wssWD}$, $GEBV_{wssPD}$) | 0.776 ($R^2$ = 0.316) | 0.567 ($R^2$ = 0.209) | 0.612 ($R^2$ = 0.217) | 0.759 ($R^2$ = 0.508) | 0.768 ($R^2$ = 0.513) |

* b – linear regression coefficient final estimates on prediction, $R^2$ – determination coefficient;
BF1—backfat thickness over 6–7 ribs, MD—muscle depth, Age—days to 100 kg, TNB—number of all piglets born at the first farrowing, NBA—number of piglets born alive at the first farrowing
